# Photo‐Assisted Charge–Discharge Behavior of NMC622 Cathode

**DOI:** 10.1002/gch2.70100

**Published:** 2026-04-10

**Authors:** Meltem Çayirli, Ersu Lökçü, Reşat Can Özden, Mustafa Anik

**Affiliations:** ^1^ Department of Metallurgical and Materials Engineering Eskisehir Osmangazi University Eskisehir Turkey

**Keywords:** chemical vapor deposition, Li‐ion batteries, N‐Doped graphene, NMC622, photo‐assistance

## Abstract

In this study, a nitrogen (N)‐doped graphene film was synthesized on copper foil via chemical vapor deposition (CVD) and employed as a photocatalytic electrode for the photo‐assisted charging and discharging of lithium‐ion batteries (LIBs). By integrating the photocatalyst as a separate electrode and allowing the cathode to function solely in its conventional role, this configuration mitigated the long‐term degradation of light‐harvesting efficiency commonly observed in semiconductor‐based cathodes and eliminated the constraint of using only semiconductor materials in LIBs. The photo‐charging response of the LIB under 1 Sun illumination yielded a photo‐conversion efficiency (η%) of 0.152% for the N‐doped graphene. Photo‐assisted operation enhanced the discharge capacity by at least 15%, improved capacity retention to 90%, and maintained a Coulombic efficiency of 100% over 100 cycles in NMC622 half‐cells. The photo‐generated electrons effectively accelerated interfacial charge transfer kinetics, thereby facilitating redox reactions at the electrode–electrolyte interface during cycling. Overall, this photo‐assisted strategy not only improves LIB performance but also offers a promising route for integrating solar energy conversion directly into energy storage technologies.

## Graphical abstract

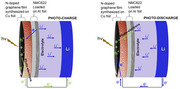

## 1 Introduction

Lithium‐ion batteries (LIBs) are widely regarded as the most promising energy storage solution for both portable electronic devices and electric vehicle (EV) applications [1, 2]. The development of various high‐capacity anode materials [3, 4, 5, 6] places significant pressure on researchers working on cathode materials, particularly to meet the demands of EVs. Layered transition‐metal oxides (LiNi_x_Co_y_Mn_z_O_2_; NCM) are considered among the most promising candidates for high‐energy‐density cathode materials [7]. However, NMC cathodes face a significant challenge known as cation mixing, which arises from the exchange of Li and Ni ions due to their similar ionic radii (Li^+^: 0.076 nm, Ni^2^^+^: 0.069 nm). This issue occurs during material synthesis and persists throughout prolonged charge–discharge cycles, leading to capacity fade and structural degradation [8, 9, 10, 11]. Numerous strategies have been employed to overcome these problems in NMC cathodes, such as surface coating [12, 13], cation doping [14, 15, 16], and nanoscale synthesis to reduce the distance of Li^+^ transport [17, 18].

Growing demand for a green energy supply has opened a novel pathway that involves the integration of solar energy conversion and storage systems. Among the various strategies belonging to this pathway, the photo‐assisted charging–discharging attracts great attention. The photo‐assisted charging of Li‐ion oxygen batteries with the aid of triiodide/iodide (I_3_^−^/I^−^) redox shuttling constitutes the major part of these studies [19, 20, 21, 22, 23, 24]. The photo‐assisted charging and discharging of the LIBs, however, holds the greatest promise due to their high operating voltage, excellent reversibility and cycle stability, as well as proven commercial success [25]. The charging–discharging behaviors of LIBs with the dual‐function photo‐electrodes like LiMn_2_O_4_ [26], V_2_O_5_/ P3HT/rGO [27], and LiV_2_O_5_ [28] cathodes under both the light‐only and biased current conditions were reported recently. All these works pointed out the remarkable performance increase in the LIBs with the photo‐assistance. The inevitable degradation in the photo‐catalyzer performance of the dual‐function cathode during the Li‐ion insertion and extraction cycles, however, remains a big problem to be solved.

In this study, N‐doped graphene was synthesized on one side of a copper foil using the CVD technique. Following the synthesis, the uncoated side of the copper foil was placed in contact with an aluminum foil, on the opposite side of which the NMC622 cathode was loaded (Graphical Abstract). This procedure separated the photo‐catalyzer and cathode, allowing both of them to show only their function. Furthermore, our design, which utilizes the graphene film directly on its copper substrate, eliminates film transfer steps that often lead to wrinkling and tearing. As an alternative strategy to enhance the performance of the NMC622 cathode in LIBs, the influence of photo‐assistance on the charge–discharge behavior of the NMC622 cathode was systematically investigated.

## 2 Experimental Section

### 2.1 CVD Synthesis

N‐doped graphene was synthesized via CVD, following the procedure outlined in Figure S1. After cleaning, a 1 cm × 1 cm copper foil substrate was placed in the hot zone of the CVD system, while urea powder (CO(NH_2_)_2_; 10 mg/cm^2^), serving as the nitrogen source, was positioned in the cold zone. During graphene growth, the urea powder was gradually drawn into the zone maintained at 60°C–75°C, and by the end of the process, it was ensured that the entire amount had fully evaporated. After the synthesis, the N‐doped graphene film on the copper foil was coated with polymethyl methacrylate (PMMA) using a spin coater. The copper substrate was then dissolved in a 50% HCl solution containing 0.5 m FeCl_3_. After transferring the PMMA‐coated graphene film onto either SiO_2_/Si (for characterizations) or ITO‐coated glass (Rs < 50 Ω/sq) (for electrochemical measurement), the PMMA layer was removed using acetone and isopropanol.

### 2.2 Electrode Preparations and Electrochemical Measurements

Photocurrent and Mott–Schottky measurements were performed in a conventional three‐electrode setup using a spectral cell filled with 0.1 m KCl buffered to pH 7 with 0.1 m K_2_HPO_4_. N‐doped graphene served as the working electrode, a platinum wire as the counter electrode, and an Ag/AgCl (saturated KCl) electrode as the reference. Measurements were carried out at 1 V_Ag/AgCl_ using a Gamry Reference 3000 workstation. A solar simulator (A‐type 150 W, 1–3 SUN, Xenon lamb, AMO filters; 400 – 700 nm wavelength) was used as the light source at both photocurrent measurements and photo‐assisted charge/discharge tests.

Charge–discharge tests were conducted using a modified 2032 coin cell with a 10 mm diameter hole (Figure S2a). The N‐doped graphene was positioned over the hole to enable light harvesting, as shown in Figure S2a. After assembly, the coin cell was placed into a custom‐designed holder (Figure S2b), with a spectral window aligned to cover the holed section of the coin cell for controlled isolation. All the test assemblages were carried out in an Ar‐filled glove box with H_2_O and O_2_ levels less than 0.1 ppm. Lithium metal was used as both the counter and reference electrodes, while polipropilen (Celgrad 2400) membrane served as the separator. The electrolyte was 1.2 m LiPF_6_:EC: DMC (4:6 by volume). The NMC622 electrode composition consisted of 80 wt.% active material, 10 wt.% Super P carbon black, and 10 wt.% polyvinylidene fluoride (PVDF) as a binder. The active electrode materials were first subjected to heat treatment at 120°C for 2 h under vacuum. After the thermal treatment, the materials were mixed with Super P carbon black in a ball mill (Fritsch, Pulverisette 7) for 30 min. The resulting mixture was slowly added to a PVDF solution completely dissolved in N‐methyl‐2‐pyrrolidone (NMP) to form an electrode slurry. This slurry was then coated onto carbon‐coated aluminum foil using a doctor blade, with a wet thickness of 100 microns. Finally, the coated electrodes were dried at 100°C under vacuum for 24 h. The galvanostatic discharge and charge test cut‐off potentials were 2.8 V_Li+/Li_ and 4.3 V_Li+/Li_, respectively. To prevent overheating during the photo‐assisted charge/discharge tests, the cell was continuously cooled with a fan, and the temperature of the spectral glass covering the cell was monitored using an infrared thermometer, remaining below a maximum of 35°C. The number of cycles was set to 100 at the selected C‐rates.

### 2.3 Characterizations

The morphology examination was carried out with a ZEISS Ultraplus scanning electron microscope (SEM). X‐ray diffraction (XRD) analyses were performed using a Panalytical Empyrean XRD instrument. A copper tube (λ = 1.5418 Å) was used as the X‐ray source. The scan was conducted in the 2θ range of 10° to 60° at a rate of 2°/min. The Raman analysis was done by Raman spectroscopy (RENISHAW RAMAN in Via Microscope) with an excitation laser of 633 nm. UV/Vis spectra were recorded using a Cary 5000 UV/Vis/NIR spectrometer equipped with a diffuse reflectance accessory, over the range of 200–800 nm.

## 3 Results and Discussions

### 3.1 Structural and Optical Characterizations

**FIGURE 1:**
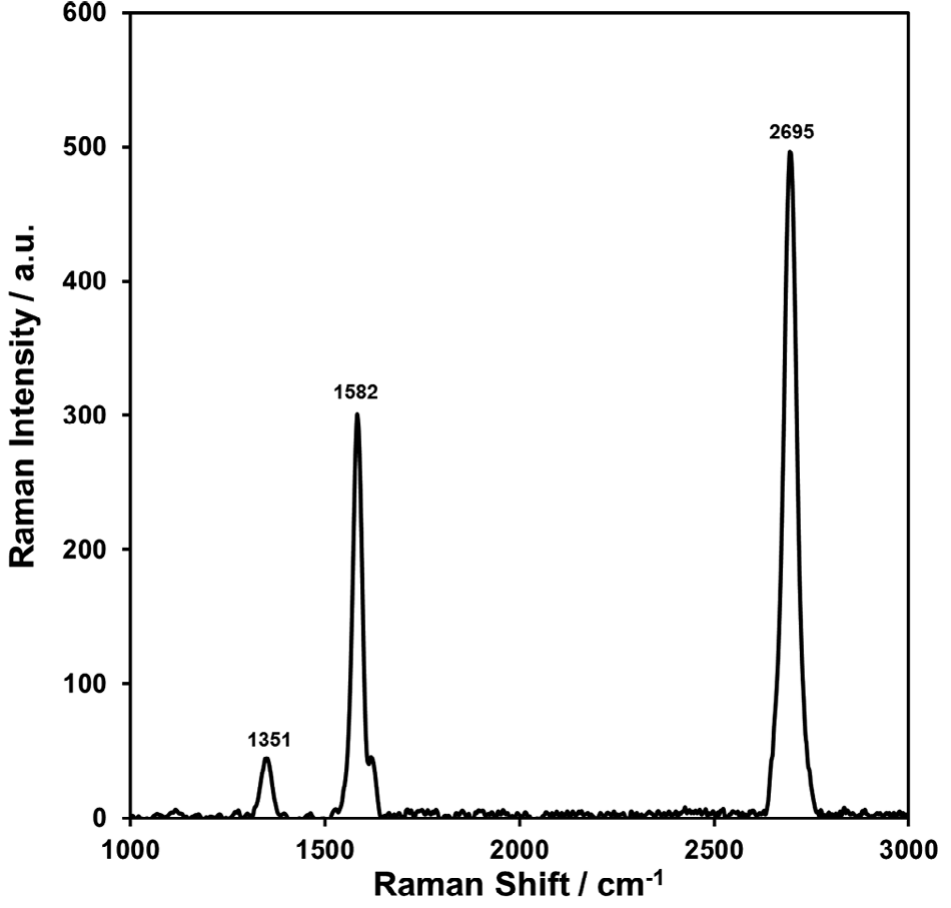
The normalized Raman spectra of the N‐doped graphene film.

The normalized Raman spectra of the graphene film synthesized with 10 mg urea (CO(NH_2_)_2_) per cm^2^ of copper substrate is provided in Figure 1. Three main peaks are observed in the Raman Spectrum. The D band (∼1351 cm^−1^) indicates the atomic doping, where the insertion of nitrogen atoms into the sp^2^ carbon matrix introduces topological defects in the N‐doped films [29]. The G (∼1582 cm^−1^) and 2D (∼2694 cm^−1^) bands are associated with the sp^2^ hybridizations of the C atoms [30]. The 2D band is sensitive to the number of layers in graphene films [30], and the 2D/G intensity ratio of 1.8 shown in Figure 1 indicates that the synthesized film consists of a few layers. The detailed SEM, AFM, TEM, and XPS characterizations of the synthesized N‐doped graphene films were provided in our previous publication [23].

**FIGURE 2:**
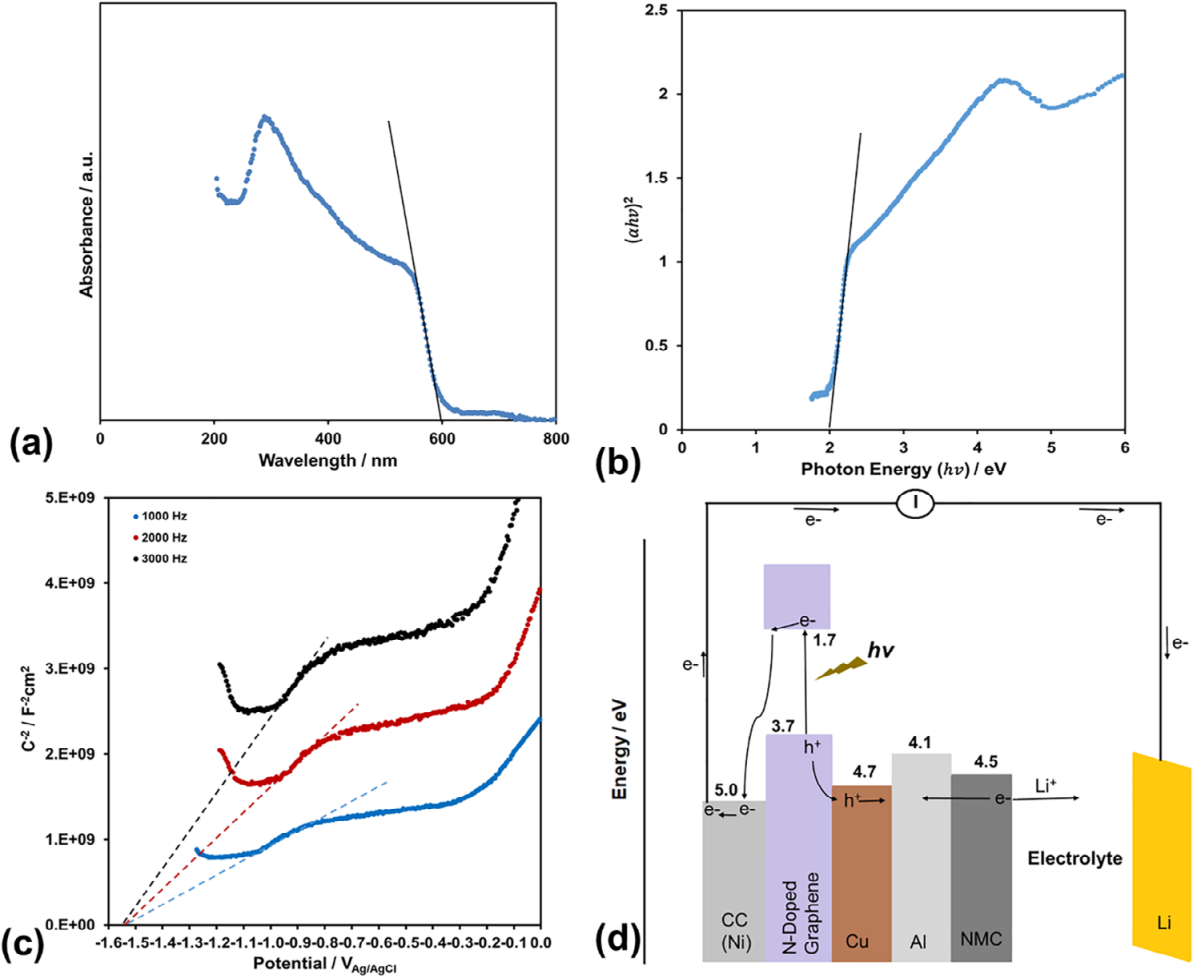
(a) Absorption band edge, (b) optical bandgap, and (c) CB edge potential of the N‐doped graphene film, and (d) energy band diagram of the N‐doped graphene film/Cu/Al/NMC622 system in Li‐ion battery.

The UV–Vis diffuse reflectance spectrum of the synthesized N‐doped graphene film is shown in Figure 2a, with an absorption band edge at 600 nm. The corresponding optical band gap (Eg) is determined to be 2.0 eV from the Tauc plot [21, 22, 23, 24] constructed using the data in Figure 2a, as shown in Figure 2b. Assuming the flat band potential to be approximately equal to the conduction band (CB) edge potential, the Mott–Schottky plot (Figure 2c) is employed to determine the CB edge. As shown in Figure 2c, the CB edge potential of the N‐doped graphene film is −1.56 V_Ag/AgCl_ (equivalent to 1.7 $V_{\mathrm{Li}/Li^{+}}$). The valence band (VB) edge potential is subsequently calculated by adding the Eg to the CB edge potential, yielding a value of 3.7 eV. Based on the calculated values, the energy band diagram of the N‐doped graphene film/Cu/Al/NMC622 system in a Li‐ion battery is presented in Figure 2d.

The photocatalytic activity of the synthesized *n*‐type N‐doped graphene film is demonstrated by the photoanodic currents shown in Figure S3. In the on‐off experiments conducted under visible light at a constant potential of 1 V_Ag/AgCl_, the semiconductor film is observed to generate a photocurrent of approximately 10^−6^ A/cm^2^.

**FIGURE 3:**
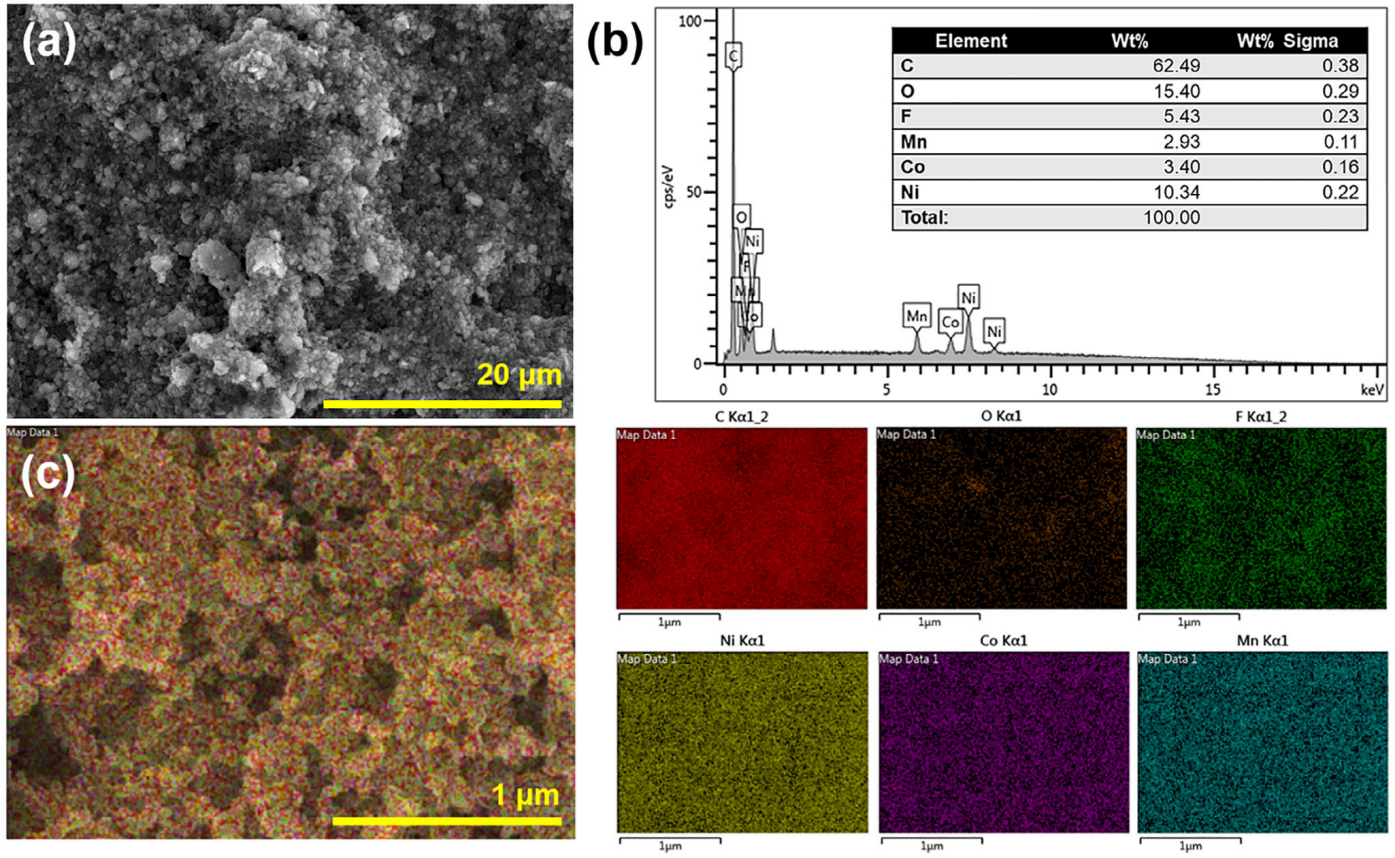
(a) SEM image, (b) EDS analysis, and (c) elemental mapping of NMC622 electrode.

**FIGURE 4:**
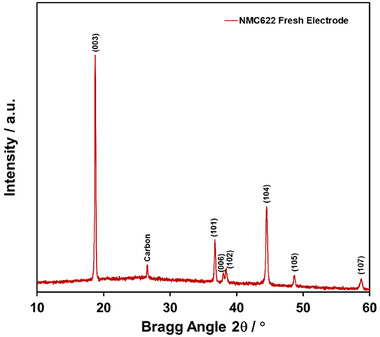
XRD of NMC622 electrode.

The SEM image of the NMC622 cathode electrode is given in Figure 3a. The evenly placed active material particles are clearly visible on the electrode surface. The EDS analysis shows that Ni:Mn:Co atomic ratios are compatible with the composition of NMC622 in Figure 3b. The elemental mapping in Figure 3c reveals that Ni, Mn, and Co are homogeneously distributed over the electrode surface. The XRD pattern of the freshly prepared NMC622 electrode on the carbon‐coated Al surface is presented in Figure 4. The intense (003) peak observed at 2θ = 19° indicates the strong crystalline structure of NMC622 [31]. A carbon peak, originating from the electrode material, is also observable. Peaks above 2θ = 60° are not shown, as reflections from the Al substrate appear at higher angles.

### 3.2 Photo‐Assisted Charge/Discharge

In the NMC composition, Ni enhances redox reactions; Mn, which is electrochemically inactive within the operating voltage range, stabilizes the cathode structure, particularly during charging; and Co facilitates redox reactions while also reinforcing the layered structure, thereby improving the rate capability. NMC622 has a nearly optimized composition. The typical charge–discharge curve of the NMC622 half‐cell at 0.1 C rate is shown in Figure S4, exhibiting an approximate discharge capacity of 190 mA h g^−1^. The charge voltage suddenly rises to 3.6 V_Li/Li+_, after which the extraction of Li from the crystal lattice begins. The charge curve has a sloping region that arises from the oxidation of Co^3+^ to Co^4+^ and Ni^2+/3+^ to Ni^4+^. During discharge, the intercalation of Li^+^ ions into the crystal lattice is accompanied by cation reductions down to a potential of 3.6 V_Li/Li+_, after which the potential drops sharply. The Coulombic efficiency in Figure S4 is at around 98%.

The charge–discharge cycling curves of the NMC622 half‐cells at 0.5 C, 1 C, 2 C, and 5 C rates are provided in Figure S5a–d, respectively. The initial discharge capacities of the NMC622 cathode at 0.5 C, 1 C, 2 C, and 5 C rates are 170, 157, 138, and 112 mA h g^−1^, respectively. At all rates over the 100 cycles, a voltage decay is observed due to the trapping of metal ions in tetrahedral sites during charge–discharge cycling [32]. As shown in Figure S5, capacity retention ranges between 75% and 80% at all rates, while the Coulombic efficiency remains above 96%, as depicted in Figure S6 by comparing the charge and discharge capacities over 100 cycles.

The SEM image of the NMC622 cathode electrode after 100 cycles is shown in Figure S7a. No visible physical damage is observed on the electrode surface. The EDS analysis (Figure S7b) and elemental mapping (Figure S7c) exhibit features nearly identical to those in Figure 3b,c, indicating no compositional degradation in the NMC622 electrode after 100 cycles. The absence of physical and compositional damage on the electrode implies that the capacity fade over 100 cycles is attributable to structural changes in the electrode, as noted previously. Figure S8 shows the XRD patterns of pristine and cycled electrodes of NMC622. The peak intensities decrease in the cycled NMC622 electrode. The intensity ratio of the (003) to (104) peaks, which depends on the amount of antisite Ni and Li, allows for the identification and quantification of cation mixing in the NMC electrodes [33]. Fundamentally, a reduction in the (003)/(104) intensity ratio indicates increased cation mixing [33]. The calculated intensity ratios, from Figure S8, for the pristine and cycled electrodes are 2.80 and 2.05, respectively, suggesting enhanced cation mixing after prolonged charge–discharge cycling (100 cycles). Despite the well‐designed composition of NMC622, structural degradation appears to be inevitable, highlighting the need for novel strategies to maintain the performance of NMC electrodes in LIB applications.

In this study, within the scope of integrating solar energy conversion and storage systems, we investigated the effect of photo‐assistance on the charge–discharge performance of the NMC622 cathode in LIBs. Prior to the photo‐assistance tests, the photo‐charging response of the LIB under 1 Sun illumination is evaluated, as shown in Figure S9. The photo‐conversion efficiency (η%) of the N‐doped graphene is calculated to be 0.152%, with calculation details provided in the Supporting Information. This efficiency surpasses that of many photo‐catalysts reported in the literature [25].

**FIGURE 5:**
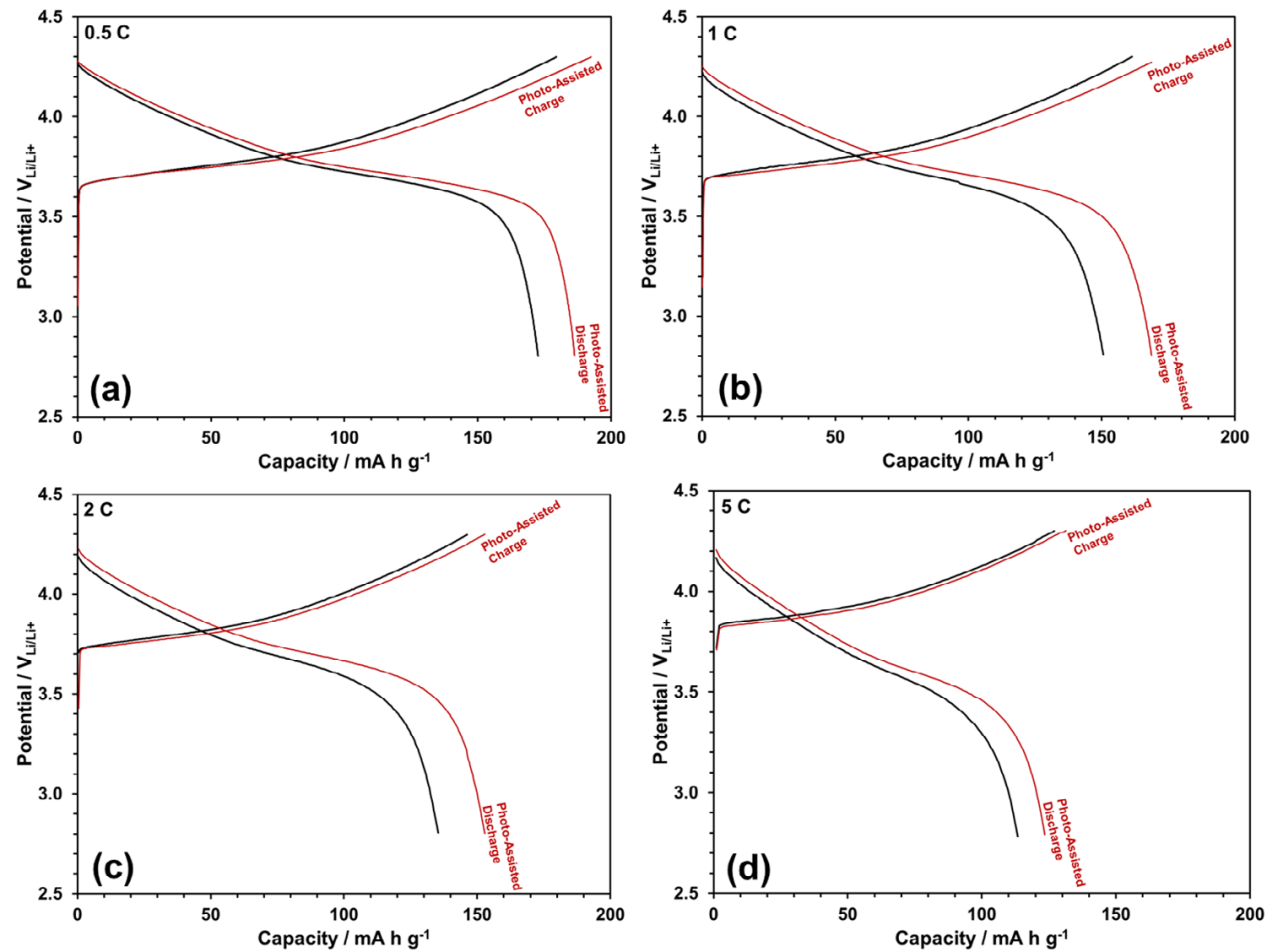
Effect of photo‐assistance on the charge–discharge curves of the NMC622 cathode at (a) 0.5 C, (b) 1 C, (c) 2 C, and (d) 5 C rates.

**FIGURE 6:**
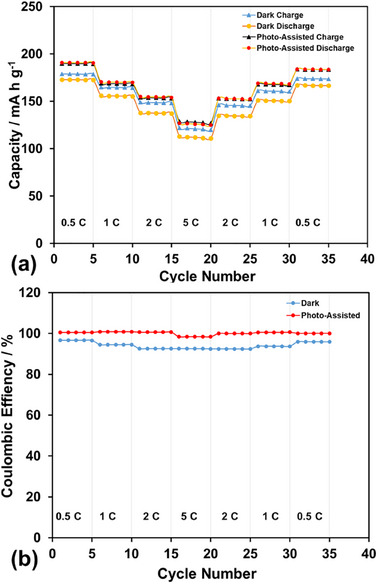
Effect of photo‐assistance on (a) the C‐rate and (b) Coulombic efficiency.

The improvements in charge–discharge capacity with photo‐assistance are shown in Figure 5a–d at the 0.5 C, 1 C, 2 C, and 5 C rates, respectively. With photo‐assistance, the charging potential decreases while the discharging potential increases, leading to an overall enhancement in both charge and discharge capacities. The effects of photo‐assistance on the C‐rate and Coulombic efficiency are depicted in Figure 6a,b, respectively. Photo‐assistance not only enhances the charge and discharge capacities but also improves Coulombic efficiency, which remains at 100% over 100 cycles, despite a slight reduction at a 5 C rate. The charge (forward reaction) and discharge (backward reaction) reactions of the NMC622 cathode are presented in Reactions 1 (cathode side) and 2 (anode side), respectively.

(1) $$\mathrm{LiN}i_{0.6}Mn_{0.2}Co_{0.2}O_{2}⇄Li_{\left(1-x\right)}Ni_{0.6}Mn_{0.2}Co_{0.2}O_{2}+\mathrm{xL}i^{+}+xe^{-}$$

(2) $$\mathrm{xL}i^{+}+xe^{-}⇄\mathrm{xLi}$$

During photo‐assistance, electrons generated in the conduction band of N‐doped graphene are transferred to the Li anode, where they participate in the reduction reaction described in Reaction (2) (Figure 2d). As a result, the charge potential of the lithium‐ion battery decreases due to compensation by the generated photovoltage. The remaining holes facilitate lithium extraction from the NMC622 cathode (Figure 2d). While the photo‐generated electrons contribute to the reduction process in Reaction (1), the holes are involved in the oxidation process described in Reaction (3) during discharge (Graphical Abstract). The generated photovoltage, in the discharge process, increases the discharge potential.

(3) $$\mathrm{xLi}+xh^{+}\to \mathrm{xL}i^{+}$$

Photo‐assistance appears to enhance the discharge capacity to a greater extent than the charge capacity in Figure 5. This is likely due to the resistance encountered by photo‐generated electrons during their transfer from the cathode to the anode in the charging process. In contrast, during discharge, the photo‐generated electrons are consumed directly at the cathode side, eliminating the need for long‐range electron transport. The more pronounced effect of photo‐assistance on the discharge process leads to an improvement in Coulombic efficiency, as in Figure 6.

**FIGURE 7:**
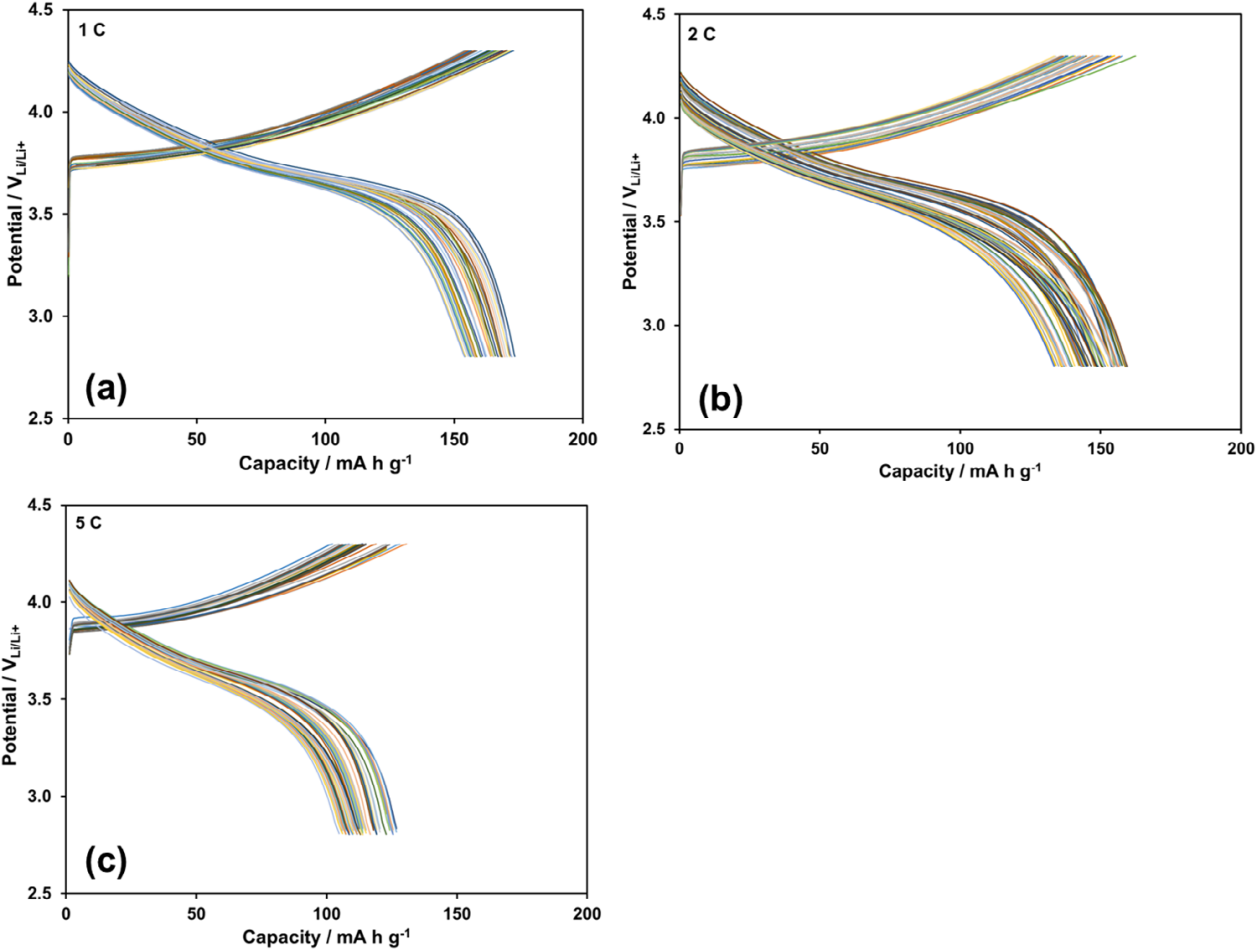
Effect of photo‐assistance on the charge and discharge curves of NMC622 half‐cell at (a) 1 C, (b) 2 C, and (c) 5 C, over 100 cycles.

**FIGURE 8:**
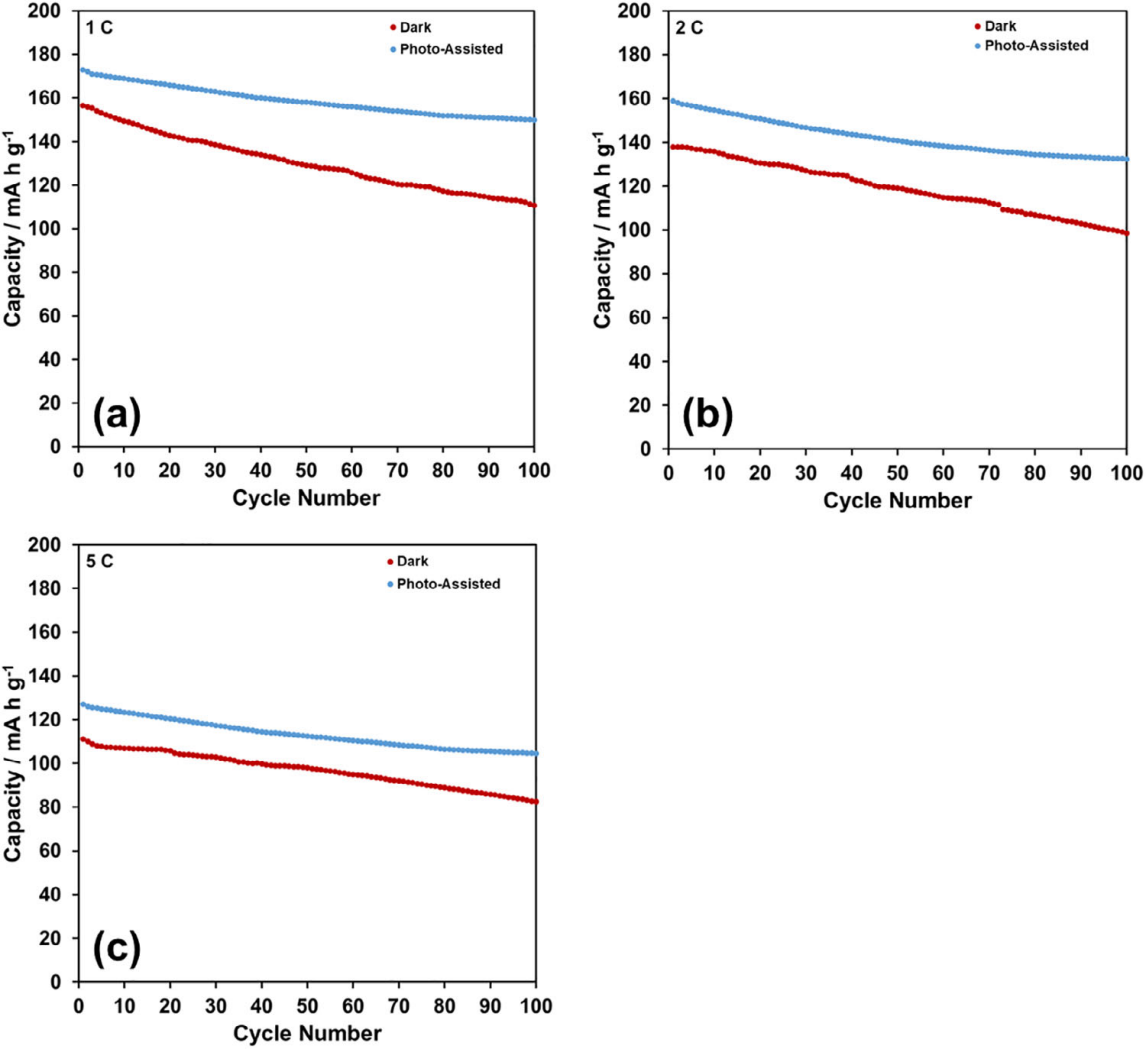
Comparison of discharge capacities of NMC622 half‐cell gathered at both dark and photo‐assisted conditions at (a) 1 C, (b) 2 C, and (c) 5 C, over 100 cycles.

The cycling performance of the NMC622 half‐cell under photo‐assistance over 100 cycles at 1 C, 2 C, and 5 C rates is presented in Figure 7a–c, respectively. To clearly illustrate the impact of photo‐assistance on LIB performance, the discharge capacities recorded under photo‐assisted conditions are compared with those obtained in the dark at the same rates over 100 cycles, as shown in Figure 8a–c, respectively. The data in Figure 8 at all current rates clearly indicate that photo‐assistance not only improves the discharge capacities but also enhances capacity retention. Under photo‐assisted conditions, the average increase in discharge capacity is 15%, with capacity retention remaining close to 90% across all C‐rates over 100 cycles. Based on the data in Figures S5 and S7, the influence of photo‐assistance on Coulombic efficiency over 100 cycles at 1 C, 2 C, and 5 C rates is presented in Figure S10. Under photo‐assisted conditions, Coulombic efficiencies exceed 100% at 1 C and 2 C, while remaining within 98.5% and 96.5% at 5 C. The effect of photo‐assistance at 5 C is less pronounced than at 1 C and 2 C. This reduced effect is likely due to the shorter illumination exposure time at higher rates, which diminishes the contribution of photo‐assistance.

The long‐term stability of the photocatalyst film is demonstrated by comparing the Raman spectra of the N‐doped graphene film in its as‐synthesized state and after 100 cycles, as shown in Figure S11. No shifts in peak positions or changes in relative peak intensity ratios are observed after 100 cycles, indicating no degradation in the photocatalyst's stability.

**FIGURE 9:**
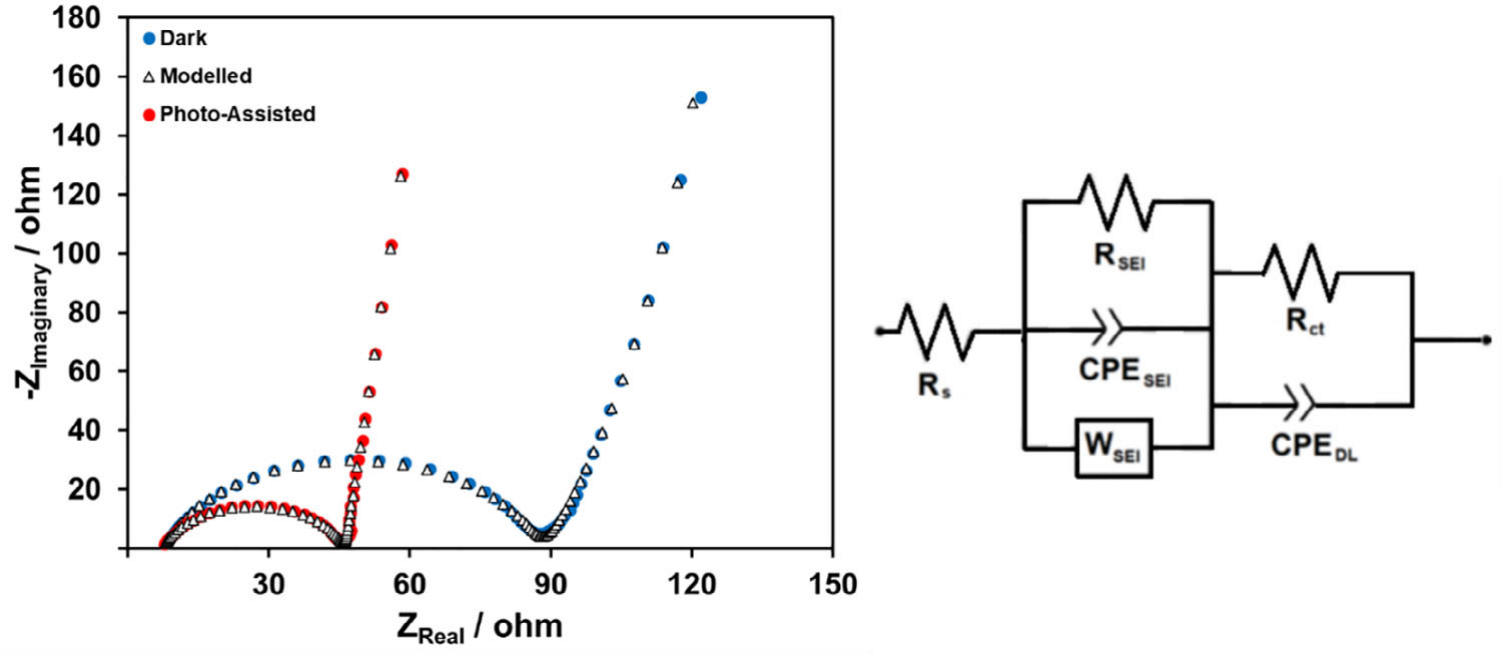
The Nyquist plots obtained at open‐circuit potential under both dark and photo‐assisted conditions for the NMC622 half‐cell. The data were fitted using the shown equivalent circuit.

Electrochemical impedance spectroscopy (EIS) is an effective technique for evaluating the electrochemical behavior of materials. The Nyquist plots obtained at open‐circuit potential under both dark and photo‐assisted conditions for the NMC622 half‐cell are presented in Figure 9. The data were fitted using the equivalent circuit shown in the same figure. The Nyquist plots exhibit two overlapping semicircles corresponding to the double layer (DL) capacitance and the solid electrolyte interphase (SEI), followed by a sloped straight line that represents Li^+^ ion diffusion. R_s_, R_SEI_, CPE_SEI_, W_SEI_, R_ct_, and CPE_DL_ stand for solution resistance, SEI resistance, SEI constant phase element, Warburg impedance, charge transfer resistance, and DL constant phase element, respectively. The charge transfer resistance (R_ct_) reflects the ease of electron transfer at the electrode surface during the charge–discharge process. A decrease in R_ct_ is generally indicative of improved electrode performance. The fitted impedance data reveal R_ct_ values of 81.28 Ω under dark conditions and 38.91 Ω under photo‐assisted conditions. The significant reduction in R_ct_ under illumination suggests that photo‐generated charge carriers effectively accelerate interfacial charge transfer kinetics, thereby facilitating the redox processes occurring at the electrode–electrolyte interface during the charge–discharge cycle.

The results of this study demonstrate that photo‐assisted charging and discharging significantly enhance the performance of NMC cathode‐based LIBs. By incorporating a photocatalyst as a separate electrode and allowing the cathode to function solely in its conventional role, this approach not only prevents the long‐term degradation of light‐harvesting efficiency seen in semiconductor‐based cathodes but also enables the use of non‐semiconductor cathode materials in LIBs. This photo‐assisted strategy offers a promising route to simultaneously improve LIB performance and integrate solar energy conversion directly into energy storage systems.

## 4 Conclusions

In conclusion, a few‐layer N‐doped graphene film was synthesized via chemical vapor deposition (CVD) to serve as a photocatalytic electrode for photo‐assisted charging of lithium‐ion batteries (LIBs). The photo‐charging response of the LIB under 1 Sun illumination provided the photo‐conversion efficiency (η%) of the N‐doped graphene as 0.152%. Photo‐assisted charge–discharge tests conducted on an NMC622 half‐cell revealed that photo‐assistance enhances discharge capacity by at least 15%, improves capacity retention to approximately 90%, and maintains Coulombic efficiency at 100% over extended cycling. The photo‐generated electrons were found to effectively accelerate interfacial charge transfer kinetics, thereby facilitating redox reactions at the electrode–electrolyte interface during the charge–discharge process. This photo‐assisted approach not only offers a novel strategy for enhancing LIB performance but also holds promise as a viable route for integrating solar energy conversion directly into energy storage systems.

## Conflicts of Interest

The authors declare no conflicts of interest.

## Supporting information




**Supporting File**: gch270100‐sup‐0001‐SuppMat.docx.

## Data Availability

The data that support the findings of this study are available from the corresponding author upon reasonable request.
