# Supplementary material for: Photo‐Assisted Charge–Discharge Behavior of NMC622 Cathode
Source: Glob Chall. 2026 Apr 10;10(4):e70100. doi: 10.1002/gch2.70100 (PMC13066908; doi:10.1002/gch2.70100)
Supplement: Supplementary file 1 — Supporting File: gch270100‐sup‐0001‐SuppMat.docx. [file GCH2-10-e70100-s001.docx]

**Supporting Information**

**Photo-Assisted Charge-Discharge Behavior of NMC622 Cathode**

Meltem Çayirli, Ersu Lökçü, Reşat Can Özden and Mustafa Anik*

Department of Metallurgical and Materials Engineering, Eskisehir Osmangazi University, 26040, Eskisehir, Turkey

*Corresponding author: [manik@ogu.edu.tr](mailto:manik@ogu.edu.tr)





**Figure S1**. The synthesis of N-doped graphene films by CVD.


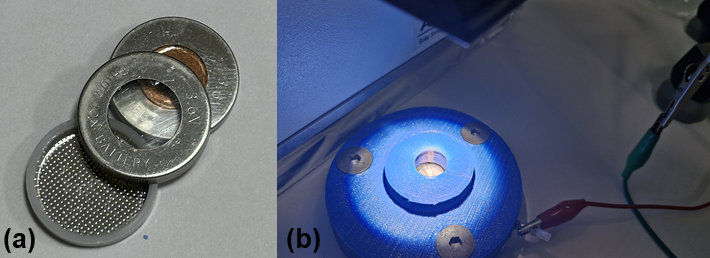


**Figure S2.** (a) A 2032 coin cell featuring a 10 mm diameter hole sealed with a thin, transparent, and heat-resistant nylon film, and (b) a custom-designed holder with a spectral window precisely aligned to the holed section of the coin cell.


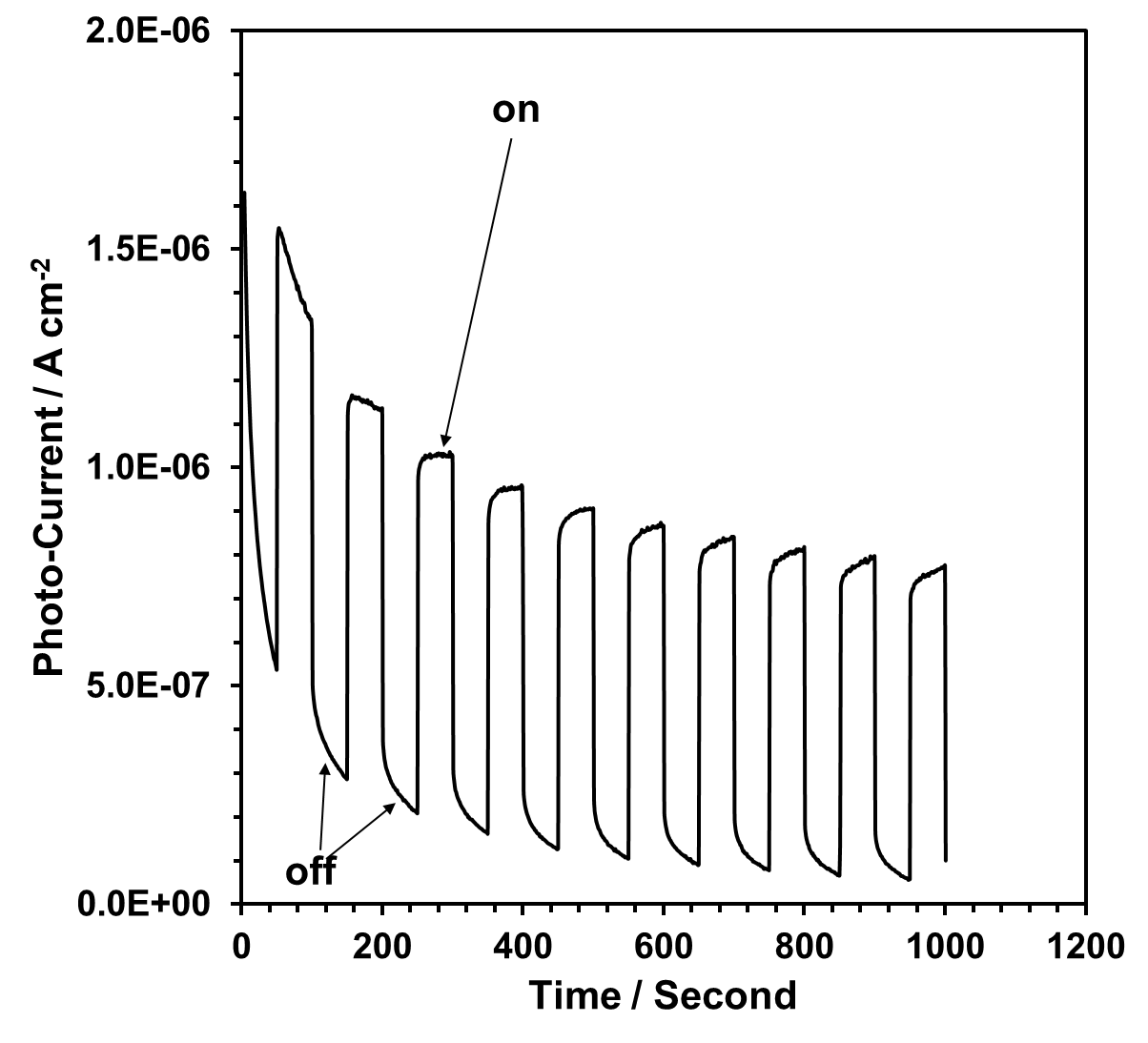


**Figure S3.** The photo-anodic currents of N-doped graphene films obtained at 1 V_Ag/AgCl_ via on-off cycles after transferring to ITO-coated glass surface.

**
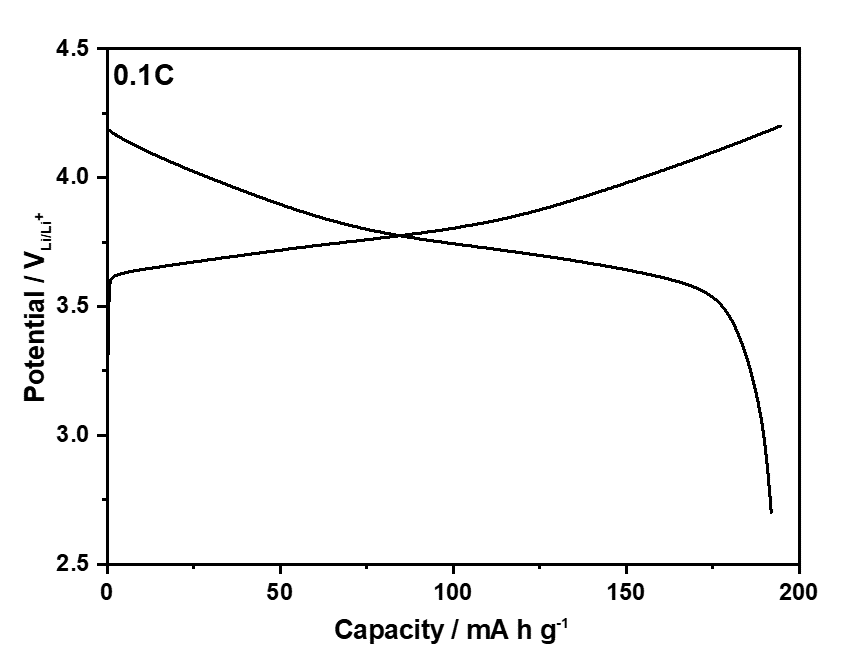
**

**Figure S4.** The charge discharge curve of NMC622 half-cell at 0.1 C.

**
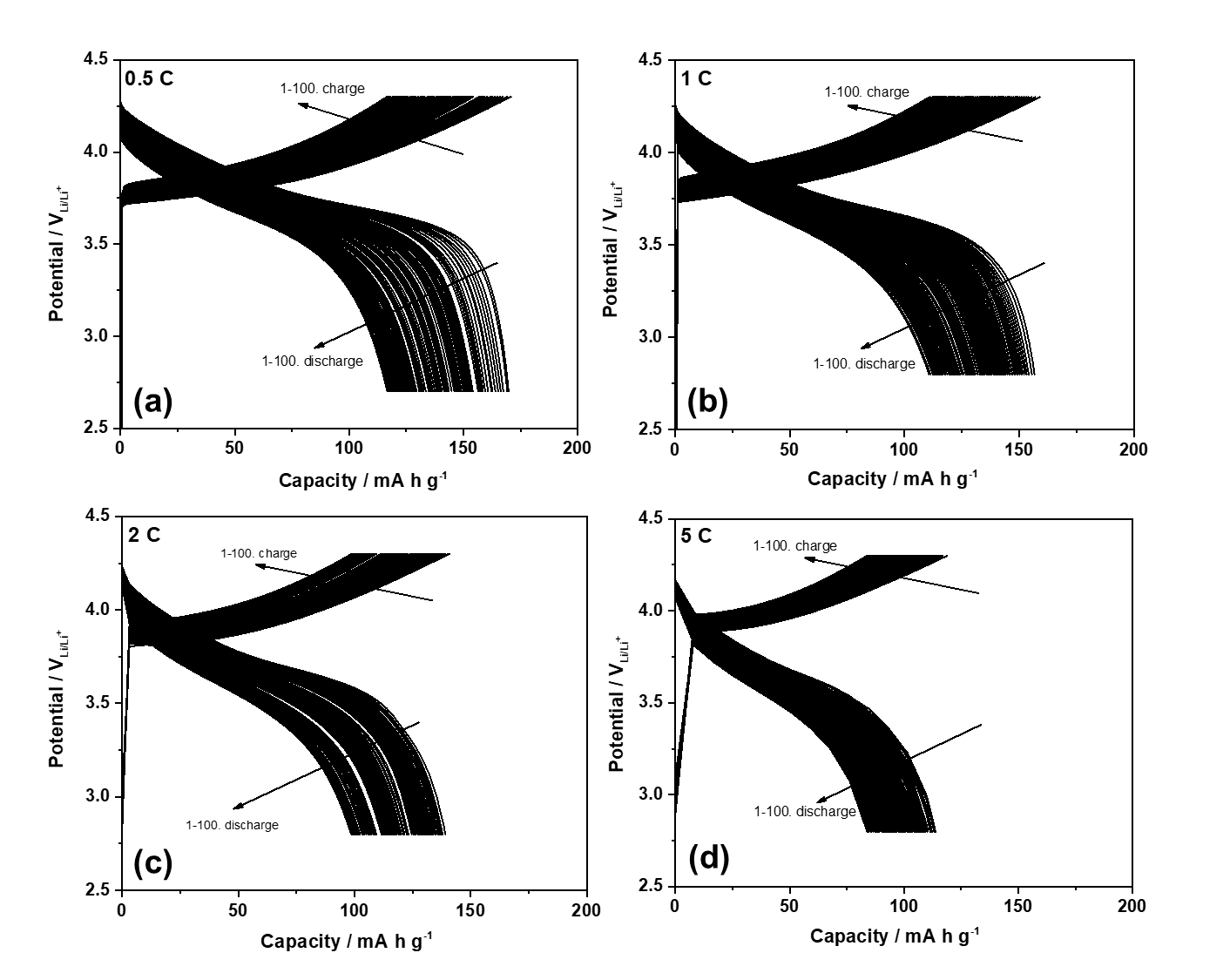
**

**Figure S5.** The 100 cycles charge–discharge curves of the NMC622 half-cells at (a) 0.5 C, (b) 1 C, (c) 2 C and (d) 5 C.

**
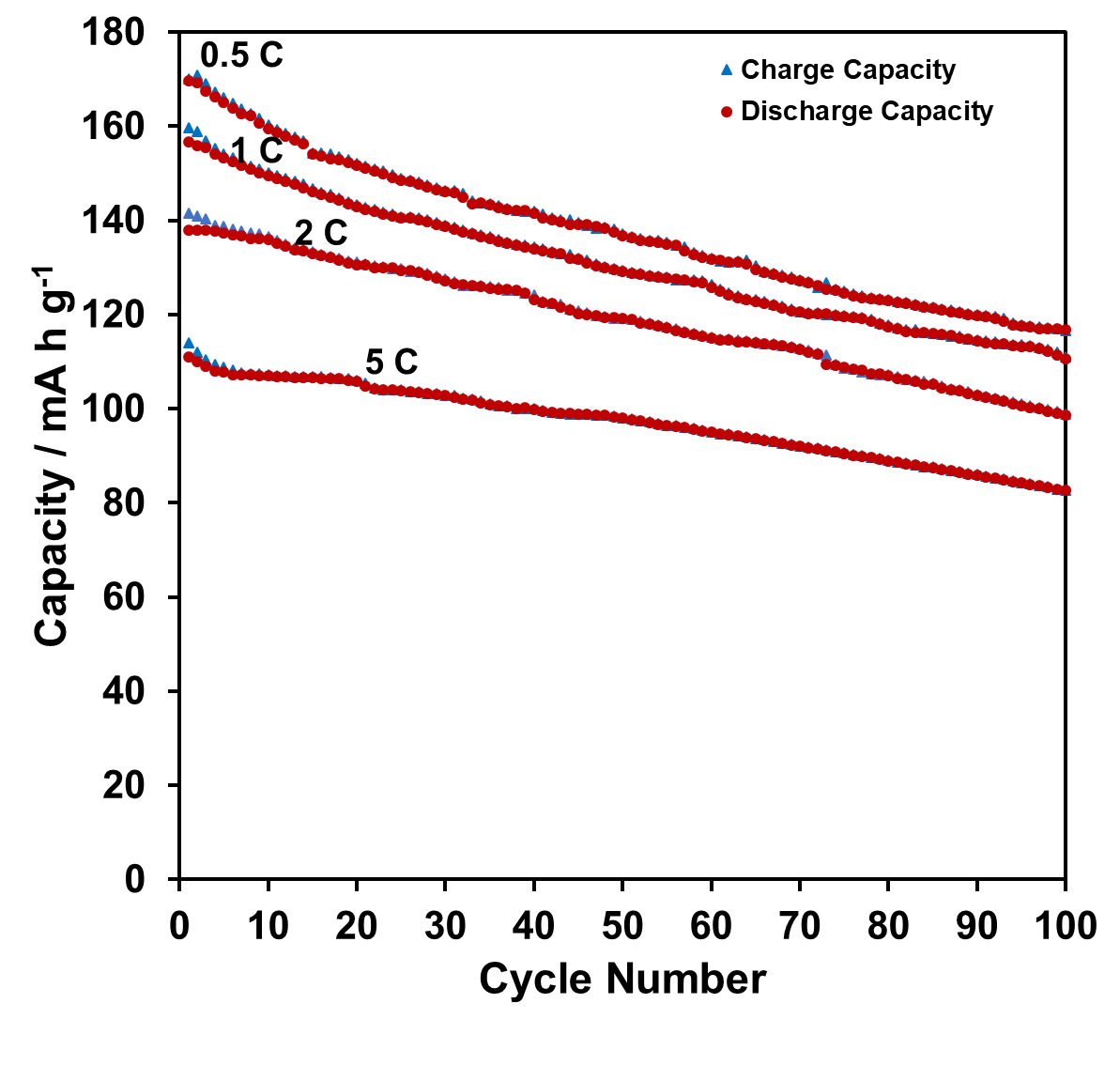
**

**Figure S6.** Comparison of charge and discharge capacities at 0.5 C, 1 C, 2 C and 5 C rates over 100 cycles for the NMC622 half-cells.

**
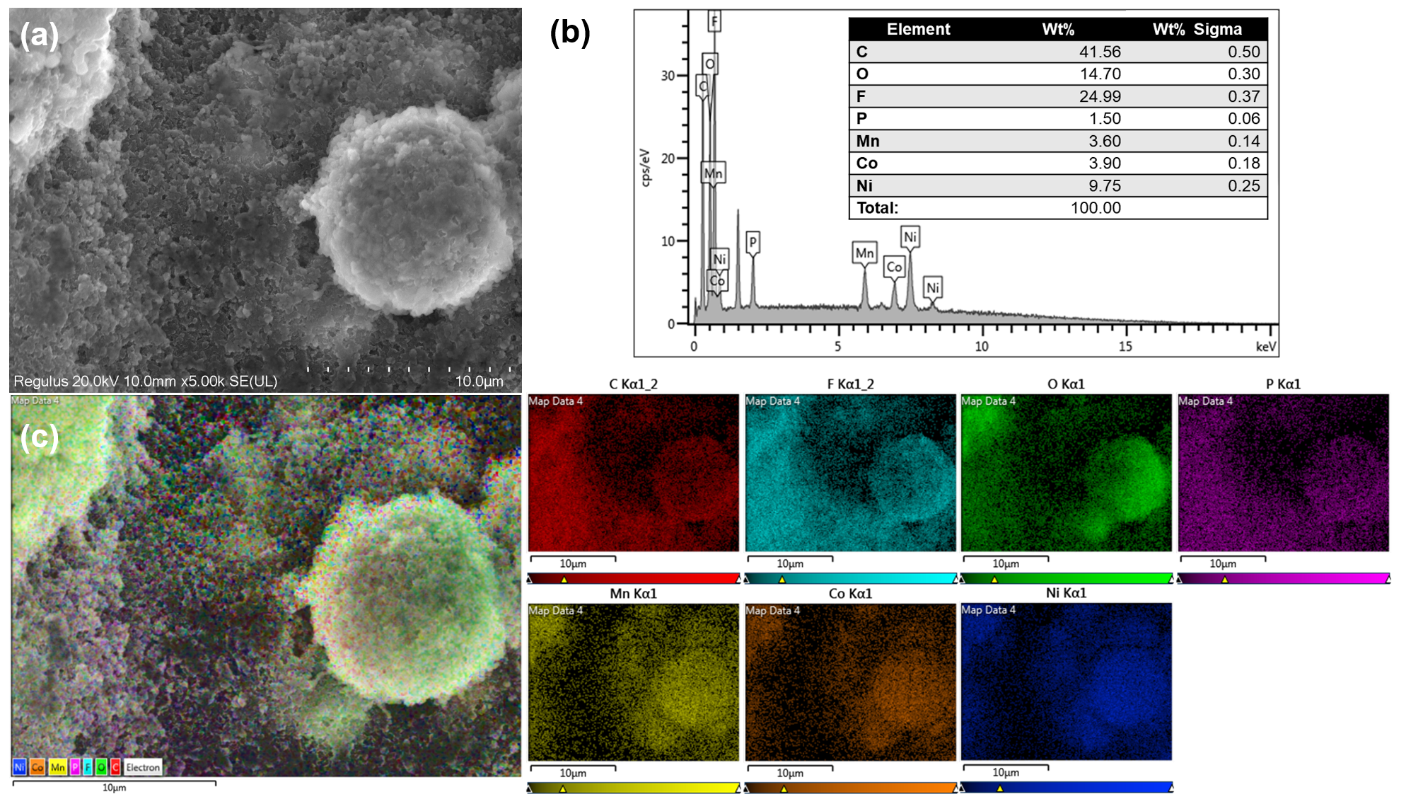
**

**Figure S7.** (a) SEM image, (b) EDS analysis and (c) elemental mapping of NMC622 electrode after 100 cycles.

**
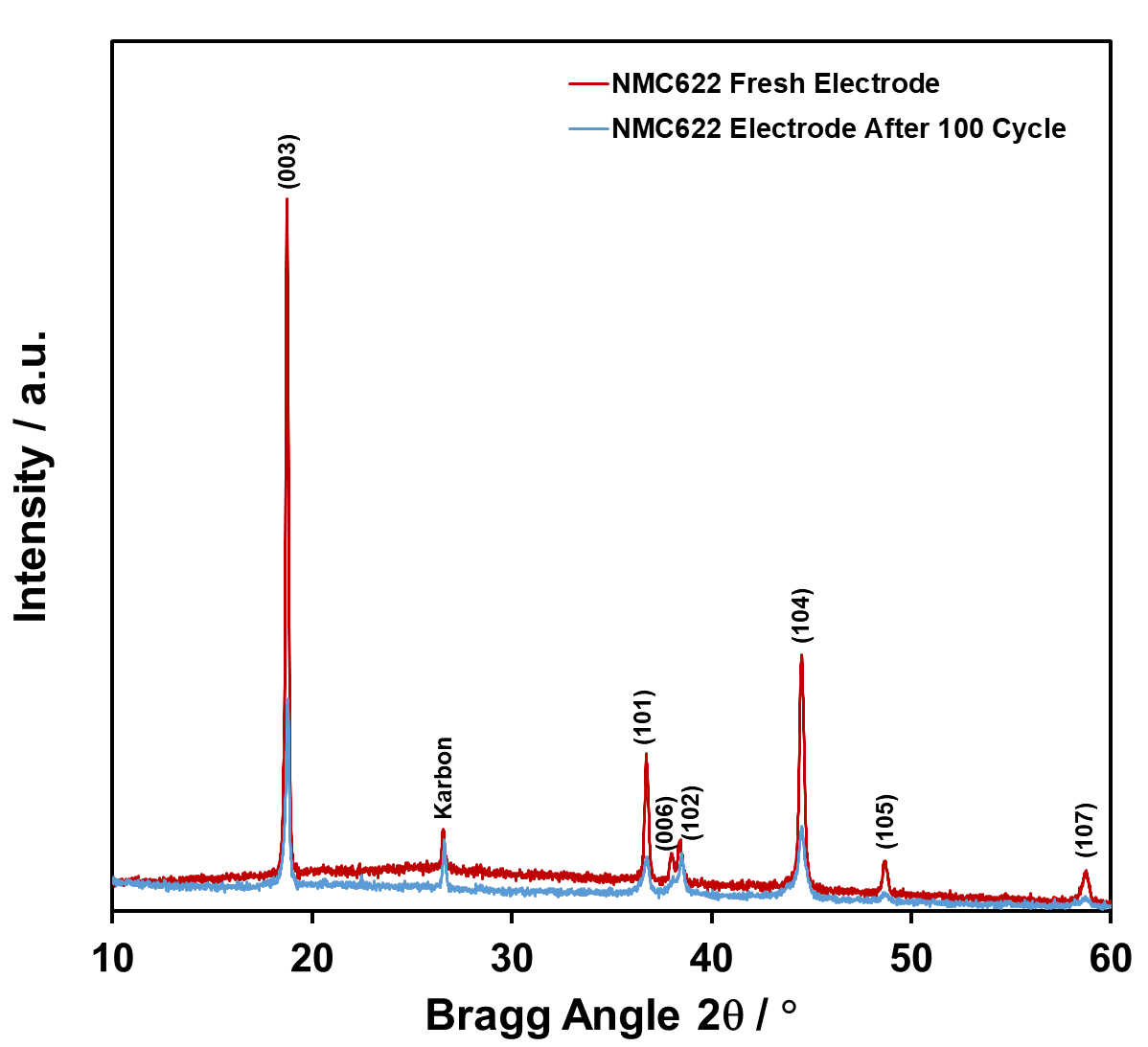
**

**Figure S8.** Comparisons of XRD of NMC622 fresh electrode with that of electrode after 100 cycles.


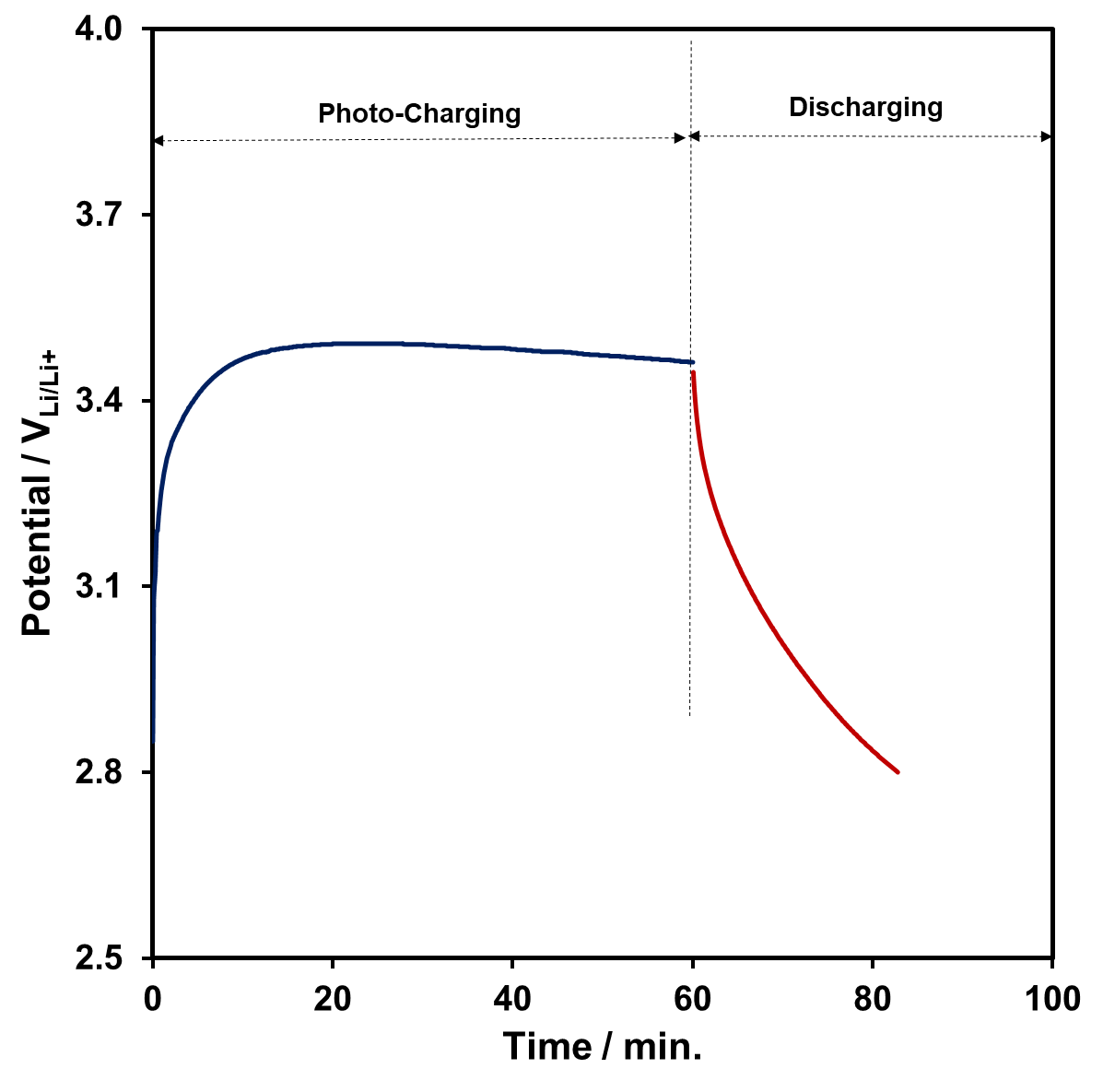


**Figure S9.** Photo-charging response of the LIB under 1 Sun illumination. Discharging current is 100 μA cm^-2^ and Gamry software provides energy output as 430 mJ (equivalent to 0.1195 mW h). Active photo-catalyzer area is 0.785 cm^2^.

**Photo-conversion Efficiency Calculation**

The photo-conversion efficiency (%𝜂) can calculated as in the following:

%𝜂 = $\frac{{Energy}_{out}}{{Energy}_{in}}$x100 = $\frac{{Energy}_{out}}{P_{in}tA}$

Energy_out_ from Figure S9 is 0.1195 mW h. P_in_ for 1 Sun illumination is 100 mW cm^-2^. Time (t) is 1 h and active photo-catalyzer area (A) is 0.785 cm^2^. Based on relationship above and the calculated parameters, the photo-conversion efficiency (%𝜂) is calculated as 0.152 for 1 Sun illumination.


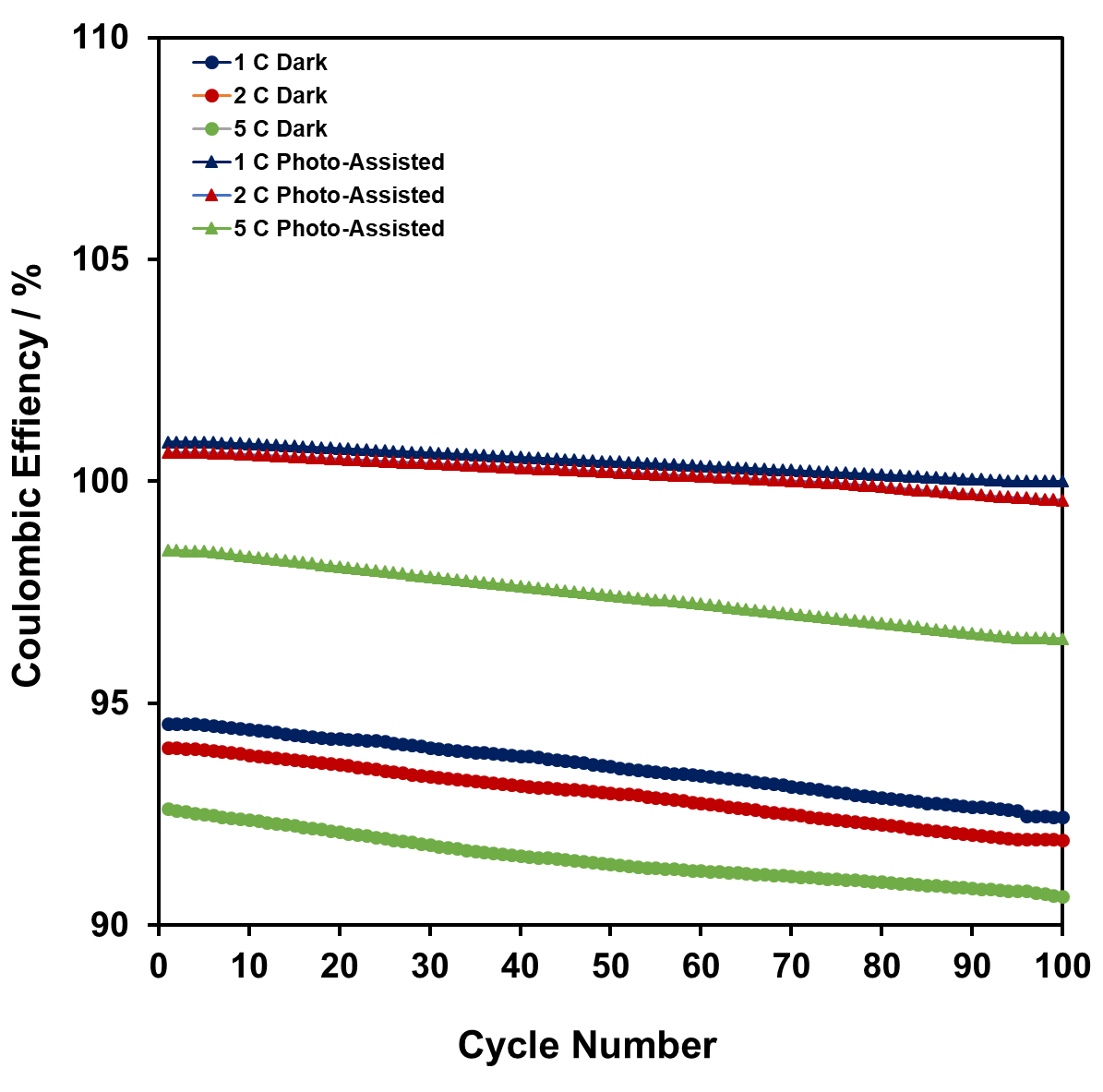


**Figure S10**. Effect of photo-assistance on Coulombic efficiencies over 100 cycles at 1 C, 2 C and 5 C rates.


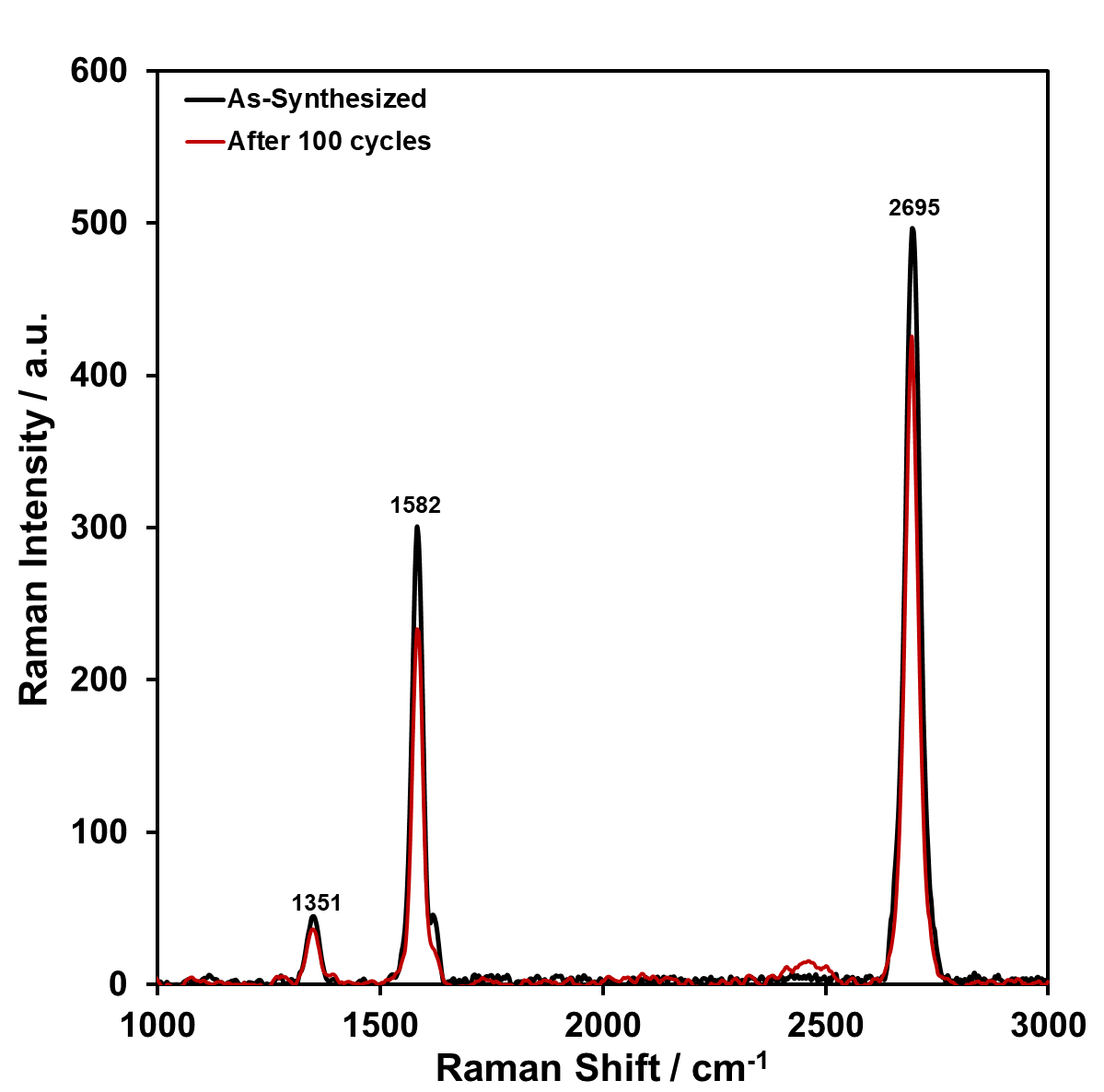


**Figure S11**. Raman spectra of the N-doped graphene film before and after 100 cycles.
